# Genetic architecture of rust resistance in a wheat (*Triticum turgidum*) diversity panel

**DOI:** 10.3389/fpls.2023.1145371

**Published:** 2023-03-14

**Authors:** Valentyna Klymiuk, Teketel Haile, Jennifer Ens, Krystalee Wiebe, Amidou N’Diaye, Andrii Fatiukha, Tamar Krugman, Roi Ben-David, Sariel Hübner, Sylvie Cloutier, Curtis J. Pozniak

**Affiliations:** ^1^ Crop Development Centre and Department of Plant Sciences, University of Saskatchewan, Saskatoon, SK, Canada; ^2^ Institute of Evolution, University of Haifa, Haifa, Israel; ^3^ Department of Vegetables and Field Crops, Institute of Plant Sciences, Agricultural Research Organization (ARO) – The Volcani Center, Rishon LeZion, Israel; ^4^ Galilee Research Institute (MIGAL), Tel Hai Academic College, Upper Galilee, Israel; ^5^ Ottawa Research and Development Centre, Agriculture and Agri-Food Canada, Ottawa, ON, Canada; ^6^ Department of Biology, University of Ottawa, Ottawa, ON, Canada

**Keywords:** wheat, leaf rust, stripe rust, stem rust, GWAS, diversity panel, *Yr15*, *Sr13*

## Abstract

**Introduction:**

Wheat rust diseases are widespread and affect all wheat growing areas around the globe. Breeding strategies focus on incorporating genetic disease resistance. However, pathogens can quickly evolve and overcome the resistance genes deployed in commercial cultivars, creating a constant need for identifying new sources of resistance.

**Methods:**

We have assembled a diverse tetraploid wheat panel comprised of 447 accessions of three Triticum turgidum subspecies and performed a genome-wide association study (GWAS) for resistance to wheat stem, stripe, and leaf rusts. The panel was genotyped with the 90K Wheat iSelect single nucleotide polymorphism (SNP) array and subsequent filtering resulted in a set of 6,410 non-redundant SNP markers with known physical positions.

**Results:**

Population structure and phylogenetic analyses revealed that the diversity panel could be divided into three subpopulations based on phylogenetic/geographic relatedness. Marker-trait associations (MTAs) were detected for two stem rust, two stripe rust and one leaf rust resistance loci. Of them, three MTAs coincide with the known rust resistance genes Sr13, Yr15 and Yr67, while the other two may harbor undescribed resistance genes.

**Discussion:**

The tetraploid wheat diversity panel, developed and characterized herein, captures wide geographic origins, genetic diversity, and evolutionary history since domestication making it a useful community resource for mapping of other agronomically important traits and for conducting evolutionary studies.

## Introduction

Three wheat rust diseases are caused by Basidiomycete fungi from the genus *Puccinia* but represent different species: stem (black) rust – *P. graminis* f. sp. *tritici* (*Pgt*), stripe (yellow) rust – *P. striiformis* f. sp. *tritici* (*Pst*) and leaf (brown) rust – *P. triticina* (*Pt*). These species are present in all wheat growing environments and can cause substantial yield losses if conditions for infection and spread are favorable and susceptible varieties are grown (Savary et al., 2019). Approaches to prevent yield losses include timely application of fungicides and elimination of rust alternative hosts (e.g., barbery species); however, the most economical, efficient, and environmentally friendly strategy to mitigate losses from these diseases is the use of resistant cultivars. Currently, 83 stem rust (*Sr*), 130 stripe rust (*Yr*) and 117 leaf rust (*Lr*) resistance genes have been identified, which are a good source for the development of resistant cultivars (McIntosh et al., 2020); however, many of them have been defeated by adapted pathogen races.

Rust races can rapidly evolve, leading to a breakdown of deployed resistance in commercial cultivars. For example, the emergence of races such as Ug99 race group or the race TKTTF has overcome many of the currently deployed *Sr* genes, which led to a broad scale infection of popular cultivars in Kenya and Ethiopia (Bhavani et al., 2022). These emergent races spread rapidly to other wheat producing regions following predicted migration paths (Singh et al., 2006). A similar situation occurred for stripe rust where novel races, adapted to diverse environments, evolved as a result of fungal sexual reproduction on an alternative host in the Himalayan region (Hovmøller et al., 2016), and are spreading rapidly across continents, leading to epidemics, e.g., outbreak in Argentina (Ali et al., 2017). In the case of leaf rust, mutations within the pathogen and subsequent selection in specific environments are the most common way for the formation of novel races (Bhavani et al., 2022). There is also evidence of intracontinental migration of leaf rust spores, similarly to that of stem and stripe rusts (Ordoñez and Kolmer, 2007). Thus, there is a need to continually search for effective rust resistance genes and to deploy them in breeding, particularly through gene pyramiding using marker-assisted selection (MAS).

Wheat relatives and landraces are known to harbor resistance genes that can be used to diversify the rust-resistance gene pool currently available for breeding. While direct use of these sources in crossing is possible, the best practice is to introduce these novel genes into adapted germplasm using marker-assisted backcrossing. However, this requires identification of tightly linked markers to select for the causal gene(s) and associated markers to minimize linkage drag and introgression of deleterious mutations. Genome-wide association studies (GWAS) are a common strategy to localize gene/quantitative trait loci (QTL) (Brachi et al., 2011). GWAS uses genotypic and phenotypic information collected on a sufficiently diverse panel to identify marker-trait associations (MTAs) and has some advantages over bi-parental QTL mapping. The most notable advantage is the use of a genetically diverse population which samples historical recombination events that have occurred over several generations, making it possible to identify QTLs with higher precision. The selection of genotypes for GWAS diversity panel should be carefully considered because mapping resolution and power are impacted by genetic diversity, relatedness within the panel, and the extent of linkage disequilibrium (LD) (Zhu et al., 2008). Most GWAS panels use diverse germplasm collections, which results in higher allelic diversity to facilitate gene discovery. Panels combining several wheat (*Triticum*) species were previously shown to be valuable for the identification of loci underlying important traits (Mazzucotelli et al., 2020; Sansaloni et al., 2020). On the downside, low frequency alleles are routinely excluded from GWAS because they do not grant sufficient statistical power to test the significance of MTAs; thus, the genes that are present at low frequencies (<5%) cannot be detected.

Several genotyping technologies are available for wheat, including simple-sequence repeat (SSR), diversity arrays technology (DArT), and single nucleotide polymorphism (SNP) markers, where the latter could be derived from exome capture (Henry et al., 2014; Govta et al., 2022), whole-genome shotgun resequencing (Chapman et al., 2015) and genotyping by sequencing (GBS) (Poland et al., 2012). SNP markers are a popular choice due to their biallelic nature, dense genome coverage, ubiquitous nature, and availability of many high-throughput detection systems, including several that have been validated for wheat, such as the wheat 9K (Cavanagh et al., 2013), 90K (Wang et al., 2014), and 820K (Winfield et al., 2016) SNP arrays.

Resistance to wheat rusts can be broadly classified into race-specific and race-non-specific resistance. Race-specific resistance is most often expressed as early as at the seedling stage (all-stage resistance (ASR)) in contrast to race-non-specific resistance, which is typically expressed late in the phenological development of the wheat plant (referred to as adult plant resistance (APR)). Generally, APR resistance is considered to be more durable because it does not create extensive selection pressure on pathogen populations. However, most breeding strategies focus on pyramiding multiple race-specific and/or race-non-specific resistance genes to increase the durability of the resistance (Stam and McDonald, 2018). At the same time, most designated rust genes are ASR with a few APRs discovered to date. Phenotyping for rust resistance can be performed in field trials or controlled environmental conditions. Field phenotyping is frequently used to assess APR, but such method requires multi-year and multi-environment trials because rust races in the field usually represent a mix of locally prevalent races. In contrast, phenotyping in controlled environments is usually performed in greenhouses or growth chambers and is typically done on seedling plants with well-characterized races. The advantages of using controlled environments for seedling phenotyping include the possibility of testing all year round, well-controlled environmental conditions to promote infection and the ability to infect plants with a single pathogen race/isolate. However, seedling phenotyping is not suited for the detection of APR resistance genes.

In the current study, we have assembled a diversity panel of 447 accessions representing three tetraploid wheat subspecies (*Triticum turgidum* 2n=4x=28): ssp. *dicoccoides*, ssp. *dicoccum* and ssp. *durum*. The panel was genotyped with the 90K Wheat iSelect SNP array (Wang et al., 2014) and was assessed for race-specific resistance following inoculation with single and highly aggressive races of stem, leaf, and stripe rust under controlled environmental conditions. These datasets were combined and used for GWAS where we detected several statistically significant MTAs – some of which overlapped with previously mapped/cloned genes, while others were detected at loci not previously reported to contain officially designated resistance genes. The SNP markers reported here will allow an exploitation of these genes to broaden the genetic base of available resistance that can be deployed in breeding to prevent losses from rust infections.

## Materials and methods

### Plant material

A diverse panel of 447 accessions representing three tetraploid wheat subspecies, including 177 accessions of wild emmer wheat (WEW; *T. turgidum* ssp. *dicoccoides*), 131 genotypes of domesticated emmer wheat (DEW; *T. turgidum* ssp. *dicoccum*), and 139 durum wheat landraces (DW_LR; *T. turgidum* ssp. *durum*) (**Supplementary Data S1**; **Supplementary Figures S1–S3**) was assembled for GWAS. Seeds were obtained from the National Small Grains Collection (USDA-ARS) through the GRIN_Global platform (https://npgsweb.ars-grin.gov/gringlobal/), International Centre for Agricultural Research in Dry Areas (ICARDA) through the Genesis platform (https://www.genesys-pgr.org/), the Lady Barbara Davis Wild Cereal Gene Bank (ICGB) at the University of Haifa, Institute of Evolution, and Agricultural Research Organization (ARO) – The Volcani Center. The geographic distribution of the genotypes was determined based on the Global Positioning System (GPS) coordinates of the collection sites, which are provided in **Supplementary Data S1** for WEW lines, or through single coordinates representing each of the countries/regions of origin for DEW and DW_LR. Visualization of the geographic distribution of genotypes from the panel was done using the GPS Visualizer browser tool. The genotypes were multiplied through single seed descent and progeny of plants that were used for genotyping and phenotyping are maintained at the Crop Development Centre, University of Saskatchewan. A set of Canadian local breeding lines and some additional genotypes were used as controls for rust inoculations (**Supplementary Table S1**).

### Rust phenotyping

#### Races

The association mapping panel was evaluated for resistance to three wheat rust pathogens. The following fungal races were selected (one per pathogen) based on their aggressiveness and virulence profile: *Pst* race W001, *Pt* race 1 (pathotype BBBD), and *Pgt* race DCB (pathotype TRRTF). *Pst* race W001 was provided by Dr. Randy Kutcher (University of Saskatchewan). *Pt* race 1 and *Pgt* race DCB were kindly provided by Dr. Brent McCallum and Dr. Tom Fetch, respectively (both from Agriculture and Agri-food Canada, Morden Research and Development Centre). To identify the virulence profile, the stem rust race was tested against the Thatcher *Sr* differential set kindly provided by Dr. Tom Fetch, that included wheat genotypes used for *Pgt* race nomenclature in North America (Roelfs and Martens, 1988). The information regarding virulence profiles of stripe and leaf rust races was retrieved from published materials (Cuomo et al., 2017; Brar et al., 2018). Virulence profiles of all three rust races are presented in **Supplementary Table S2**. The pathotypes for leaf and stem rust races were identified based on a previously described nomenclature systems (Roelfs and Martens, 1988; Long, 1989; Jin et al., 2008).

#### Experimental design and growth conditions

The entire diversity panel could not be phenotyped simultaneously because of differences in the growth rate between the wild (WEW) and domesticated (DEW/DW_LR) collections. Thus, for stem rust phenotyping the diversity panel was divided into two sets: WEW and DEW+DW_LR; while for stripe rust and leaf rust phenotyping, it was divided into three sets: WEW, DEW and DW_LR using augmented experimental designs. Five to six seeds of each genotype were sown in single 5×5×5 cm cells of 50-cell trays well-spaced out from each other. Multiple checks (**Supplementary Table S1**) were also replicated and randomly placed among the trays within each set. Trays were watered and placed at 10°C for five days to promote even germination before being returned to the growth chamber maintained at 18h/6h light/dark photoperiod and 23°C/18°C temperature. Plants were inoculated at the two-leaf stage following the procedures described below for the three rusts.

#### Leaf rust

Urediniospores were suspended in mineral oil (VWR, Canada, catalog number 470301) and applied on seedlings at the two-leaf stage using an air brush compressor and air brush kit (Mastercraft, Canada, Toronto). Plants were allowed to dry at room temperature for 1.5-2 h, before being transferred to a misting chamber providing 99% humidity at 16°C in the dark for 24 h. Then the program was changed to 70% humidity at 20°C with a light intensity of 150–170 µmol m^-2^ s^-1^ for 16 h, followed by 8 h of darkness at 16°C until the end of experiment. Plants were rated at 11 days post inoculation (dpi) using a 0 to 4 infection types (IT) scale (Stakman et al., 1962), where; 0 and 1 IT scores represent resistance, 2 IT score – moderate resistance, 3 IT score – moderate susceptibility, 4 IT score – susceptibility.

#### Stem rust

The inoculation procedure was similar to that of leaf rust except that the misting chamber was programmed to achieve 99% humidity at 20°C for 17 h in the dark followed by 4 h of light. Then, the program was changed to 70% humidity at 20°C with a light intensity of 150–170 µmol m^-2^ s^-1^ for 16 h, followed by 8 h of darkness until rating, which was performed at 11 dpi using the same IT rating scale described above for leaf rust.

#### Stripe rust

The inoculation procedure for stripe rust was as previously described (Klymiuk et al., 2022). In brief, urediniospores were suspended in mineral oil and applied to seedlings at the two-leaf stage using an air brush compressor and air brush kit. Inoculated plants were first placed in a misting chamber with 100% humidity at 10°C at 16h/8h dark/light conditions for the first 24 h after inoculation. Plants were then transferred to a growth chamber with 70% humidity at 15°C with a light intensity of 150-170 µmol m^-2^ s^-1^ for 16 h, followed by 8 h at 10°C in darkness until plants were rated at 14 dpi. Stripe rust severity was evaluated using a 0 to 9 IT scale (McNeal et al., 1971), where 0-3 IT scores represent resistance, 4-6 IT scores – moderate resistance, 7-9 IT scores – susceptibility.

### Phenotypic data analysis

Before analysis, the leaf and stem rust phenotypic data were converted from the qualitative Stakman scale (Stakman et al., 1962) into a linear quantitative scale ranging from 0 to 9 according to Zhang et al. (2011). Stripe rust IT scores were not subjected to conversion. The resulting phenotypic data was used to estimate the best linear unbiased prediction (BLUP) values for each genotype using a mixed linear model (MLM) in the R-package lmer4 (Bates et al., 2015). Checks were considered as a fixed effect, while sets, genotypes and their interactions were considered random. The “set” factor had two levels (WEW and DEW+DW_LR) for stem rust and three levels (WEW, DEW, and DW_LR) for stripe and leaf rusts.

### Genotyping and SNP filtering

Young leaf tissue was sampled at the 1-2 leaf stage. DNA was extracted and purified using the CTAB method (modified from Procunier et al., 1990). An approximate DNA concentration was estimated by agarose gel electrophoresis and DNA was normalized into a working stock of ~50ng/µl using the estimates from the gel. Genotyping was performed using the Infinium iSelect HD 90K wheat array (WG-401-1004. Illumina, San Diego, CA, United States) on the iScan instrument (Wang et al., 2014). Genotype calling was performed for the entire collection using the default genotyping module in GenomeStudio software v2.0.4 (Illumina). SNP markers with >5% missing data, <5% minor allele frequency (MAF) and monomorphic scores were removed. Additionally, SNP markers showing residual heterogeneity within an accession were converted to missing data. SNPs were positioned onto the Svevo v1 assembly (Maccaferri et al., 2019) as previously described (Fatima et al., 2020) and the resulting SNPs with known physical positions were extracted for further analysis (**Supplementary Dataset**) (Klymiuk et al., 2023). In addition, the entire diversity panel was genotyped for the presence of *Yr15* gene using *WTK1_Kin1* marker (Klymiuk et al., 2019) and *Sr13* gene using *CNL13 F/R* marker (Zhang et al., 2017).

### Linkage disequilibrium

LD analysis was performed for each chromosome by computing r^2^ values for all pairwise marker comparisons using a sliding window size of 50 markers in the software TASSEL v3.0 (Bradbury et al., 2007). The physical positions of markers were then used to estimate LD decay along each chromosome and across the entire genome by plotting the r^2^ values of loci against their physical distance (bp). LD decay was determined by fitting a smooth non-linear regression line (Marroni et al., 2011), with a critical r^2^ threshold set at the half decay distance (Hill and Weir, 1988). The intersection of the regression line with the baseline at the critical value of r^2^ was considered as the estimate of the extent of LD in the population.

### Population structure analysis

Population structure was evaluated using a model-based Bayesian clustering, a distance-based hierarchical clustering, and a principal component analysis (PCA). A model-based Bayesian clustering was conducted using the program STRUCTURE v2.3.4 (Pritchard et al., 2000), based on a subset of 2,205 weakly correlated SNPs, i.e., with squared pairwise correlations smaller than 0.2. Markov chain Monte Carlo cycles were repeated 50,000 times after 10,000 cycles of a burn-in period. The default setting of the admixture model and correlated allele frequencies was tested with the number of subpopulations (K) from two to ten. Each test included ten independent runs. Optimal K was estimated based on the ΔK – that is the rate of change in the log-likelihood of data between consecutive K values. ΔK was estimated using STRUCTURE HARVESTER, v0.6.94 (Earl and vonHoldt, 2012). Data from the ten independent runs were integrated using the FullSearch algorithm in CLUMPP v1.1.2 (Jakobsson and Rosenberg, 2007) and plotted using STRUCTURE PLOT v2.0 (Ramasamy et al., 2014). For hierarchical clustering, a dissimilarity matrix was calculated from the marker data based on Euclidean distance using the function ‘dist’ in R. Hierarchical clustering was applied to the Euclidean distance matrix based on Ward’s criterion (ward.D2) using the function ‘hclust’ in R. PCA was performed on the marker data using the function ‘svd’ in R.

### Genome-wide association analysis

Preliminary association mapping was performed based on the 6,410 physically mapped SNP markers and was conducted using a mixed linear model (MLM) (Yu et al., 2006) and a multiple locus mixed linear model (MLMM) (Segura et al., 2012). The MLM and MLMM models both accounted for population structure and pairwise relatedness or kinship as covariates. Population structure was accounted by the population membership coefficients (Q-matrix) obtained from STRUCTURE. A kinship-matrix was computed from the marker data using the software TASSEL v3.0 (Bradbury et al., 2007). MLM and MLMM analyses were performed using the software GAPIT v3 (Wang and Zhang, 2021). Preliminary analysis showed significant associations with the previously reported *Yr15* and *Sr13* physical regions. Thus, genotypic data from their gene-specific markers (*WTK1_Kin1* and *CNL13*, respectively) were added to the final analysis, which included 6,412 SNP markers (6,410 mapped SNP markers plus *Yr15* and *Sr13* markers). Analysis was conducted using the MLMM model because it is more statistically powerful than MLM and uses forward-backward stepwise linear mixed-model regression to include associated markers as covariates (Segura et al., 2012). Associations were declared significant based on the Bonferroni-corrected threshold of α = 0.05/*n*, where *n* is the number of markers.

### Alignment of quantitative trait nucleotides (QTNs) with previously reported rust resistance genes

Previously reported resistance genes were positioned onto the Svevo v1 genome assembly (Maccaferri et al., 2019) by performing basic local alignment search tool (BLAST) searches using the cloned gene coding sequences or the flanking genetic marker sequences as queries. The position of several previously reported QTLs for leaf rust resistance were included in the analysis following the same strategy (Aoun et al., 2016; Gao et al., 2016; Sapkota et al., 2019; Fatima et al., 2020). The confidence intervals for the identified QTLs were established using the genome-wide LD decay (r^2^) of 0.23 (=945,678 bp), as described in the Results section, on either side of the peak of the QTN. As such, a QTN was considered to be associated with a potentially new resistance gene when no known resistance gene(s) were positioned within the identified QTN window. Candidate genes from the confidence intervals were extracted from the annotations of the Svevo v1 (Maccaferri et al., 2019) or Zavitan v2 (Zhu et al., 2019) assemblies. Conserved domain searches were performed using the National Center for Biotechnology Information (NCBI) on-line tool “Structure” (https://www.ncbi.nlm.nih.gov/Structure/cdd/wrpsb.cgi).

## Results

### Selection of genotypes for the diversity panel

We assembled a large tetraploid wheat diversity panel that comprised accessions from three wheat *T. turgidum* subspecies (WEW, DEW and DW_LR). The genotypes were selected to represent broad geographic origin and availability in public seed banks and other collections (**Supplementary Figures S1–S3**). WEW is a collection of tetraploid accessions representing a wild progenitor gene pool of wheat, which grows naturally in a discontinuous arc of the Fertile Crescent region (Özkan et al., 2011). In our diversity panel, WEW is represented by 177 accessions collected from geographically diverse regions of seven countries (Turkey, Israel, Syria, Lebanon, Jordan, Iran, and Iraq) spanning the entire Fertile Crescent (**Supplementary Figure S1**). DEW is a collection that includes ancient non-shattering wheat genotypes that have since spread around the globe (Luo et al., 2007). The DW_LR collection harbors locally adapted cultivated genotypes that have a distinct identity, historical origin, and were not subjected to formal crop improvement (Villa et al., 2005). We selected 131 DEW accessions from 27 countries and 139 DW_LR accessions from 37 countries to represent these *T. turgidum* subspecies (**Supplementary Figures S2, S3**).

### Analysis of the SNP marker dataset and linkage disequilibrium decay

The diversity panel was genotyped using the wheat 90 K iSelect Wheat SNP array (Wang et al., 2014). Filtering removed monomorphic markers, markers with missing data and 3,757 markers with MAF <5%, producing a set of 6,473 SNPs, of which 6,410 could confidently be assigned a physical position in the Svevo v1 wheat genome assembly (Maccaferri et al., 2019). The number of markers per chromosome ranged from 205 (chromosome 4B) to 745 (chromosome 2A). The greatest proportion (~64%) of polymorphic, anchored markers resided in the A sub-genome. Markers were evenly distributed along all chromosomes, with a few regions of low density in the pericentric regions of chromosomes 3B, 4A, 4B and 6B (**Supplementary Figure S4**).

**Figure 1:**
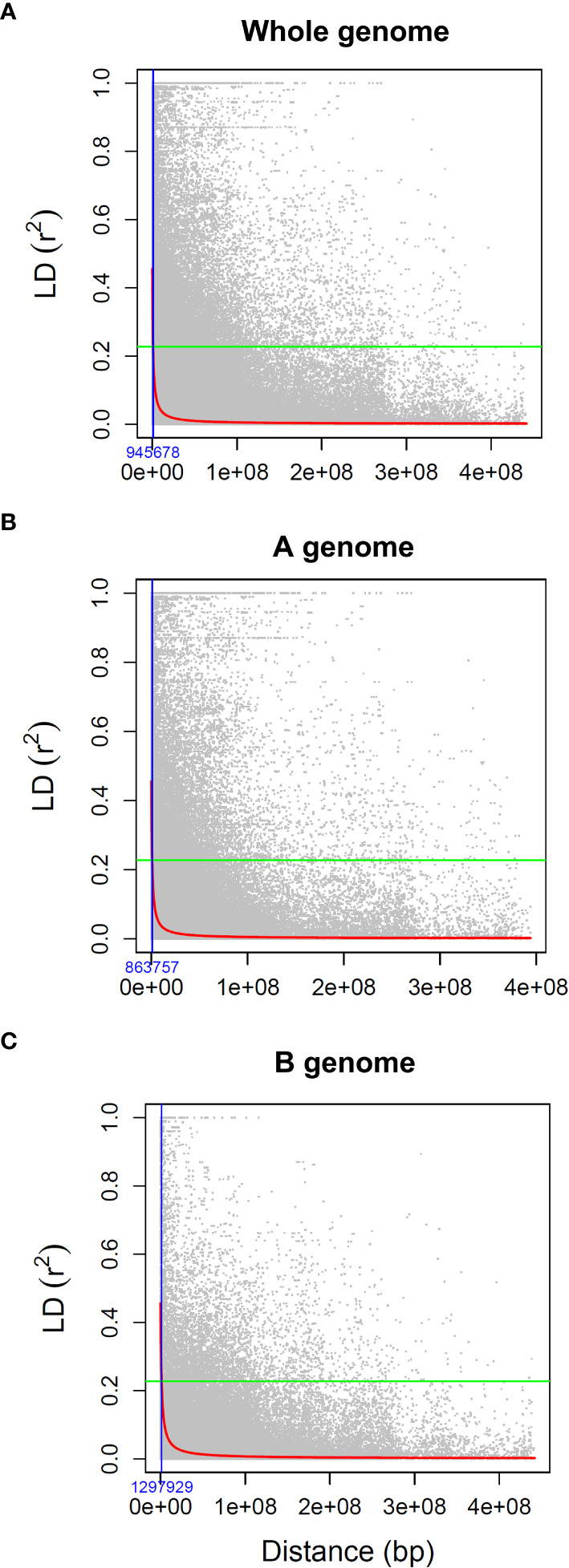
Decay of linkage disequilibrium (r^2^) as a function of physical distance (bp) between pairs of loci on **(A)** all chromosomes, **(B)** A sub-genome, and **(C)** B sub-genome. The green horizontal lines define the nominal critical levels of r^2^ = 0.23 while the fitted curve (red line) indicates the LD decay. The blue vertical line represents the extent of LD decay at the intersection of the critical r^2^ with the fitted curve.

The LD statistic (r^2^) was used to estimate the average LD decay distance in the association panel. On a genome-wide level, 10.4% of all pairs of marker loci were in significant LD (r^2^>0.2), and the average r^2^ was 0.08. The LD decay trend, determined by plotting the pairwise r^2^ values against their physical distances (**Figure 1**), revealed that the genome-wide LD decay, based on the nominal critical levels of r^2^, was 0.23 and extended to 945,678 bp (**Figure 1A**). LD decayed faster in the A genome (863,757 bp) compared to the B genome (1,297,929 bp) (**Figures 1B, C**). The genome-wide estimate of 945,678 bp distance on either side of the QTNs was used to establish confidence intervals for the QTL-harboring regions (**Figure 1**).

### Genetic diversity and population structure

**Figure 2:**
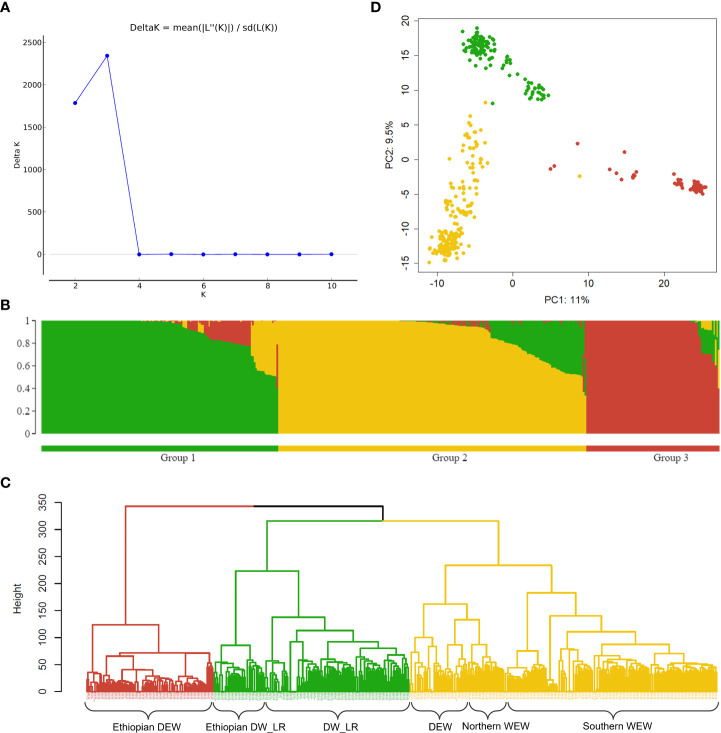
Analysis of population structure using 2,205 SNP markers. **(A)** Line graph of ΔK over K for K=2 to 10. The highest peak was observed at K = 3, which suggests three optimal subgroups. Following this, the population is divided into three color-coded subgroups: green – group 1 (DW_LR), yellow – group 2 (WEW and 41 DEW) and red – group 3 (Ethiopian DEW) represented in **(B)** Bayesian clustering, **(C)** distance-based Ward hierarchical clustering and **(D)** principal component analysis.

Analysis of population structure showed a ΔK peak at K=3, supporting three as the most probable number of subpopulations for this diversity panel (**Figure 2A**). Bayesian clustering, distance-based Ward hierarchical clustering and principal component analysis were all in agreement with this number of subpopulations which were assigned as follows: 1) DW_LR, 2) WEW + 41 DEW accessions, and 3) Ethiopian DEW (**Figure 2**; **Supplementary Data S2**). All 139 DW_LR from the diversity panel clustered in a single clade (Group 1), but the Ethiopian DW_LRs were most distinct and formed a separate subclade (**Figure 2**; **Supplementary Data S2**). A similar observation was made for 90 DEW accessions that comprised mainly Ethiopian accessions that were sufficiently divergent from the remaining DEW such that they clustered into a third clade (Group 3) (**Figure 2**; **Supplementary Data S2**). Group 2 consisted of 218 lines and included all 177 WEW lines and 41 DEW lines from broad geographic origins (**Figure 2**; **Supplementary Data S2**). A portion of 16 DEW from Group 2 clustered with the Northern WEW populations from Turkey, Iraq, and Iran.

### Phenotypic variation for rust resistance

Responses to inoculation varied within the whole collection for the three wheat rusts. A total of 68.6% of the 447 genotypes of the collection had IT≥7 and were susceptible to stem rust race DCB (**Supplementary Figure S5**). The opposite was true for leaf rust where 31.8% were susceptible (IT≥7) to race 1 (**Supplementary Figure S5**). For stripe rust, 50.1% of the genotypes were susceptible (IT≥7), 30.1% were resistant (IT ≤ 3) and 19.8% showed moderate resistance (IT>3 and <7) to W001 race (**Supplementary Figure S5**).

*T. turgidum* subspecies WEW, DEW, and DW_LR responded differently to the rust species and races. For stripe rust, all groups had a similar proportion of resistant genotypes (ranging from 25 to 36%) (**Supplementary Table S3**). Interestingly, the majority of resistant DEW genotypes were of Ethiopian origin (**Supplementary Table S4**). In response to leaf rust, the DEW collection had the highest proportion of resistant genotypes (76%), while the WEW collection contained approximately three-fold fewer resistant accessions (24%) (**Supplementary Table S3**). The Ethiopian DEW collection included higher number of accessions with resistance to race 1 (87% of resistant genotypes) compared to the rest of the DEW collection (57%) (**Supplementary Table S4**). Resistance to stem rust ranged from 2% in the WEW to 44% in the DEW collections (**Supplementary Table S3**). Accessions of the two DEW subgroups did not differ in response to stem rust, with 38% of resistant genotypes for the Ethiopian subgroup and 53% of resistant genotypes for the rest of the DEW, which contrasted phenotypic reactions to stripe and leaf rusts (**Supplementary Table S4**).

### Marker-trait associations

GWAS was performed using BLUP values for each genotype estimated based on phenotypic data obtained after inoculation with *Pgt*, *Pst* and *Pt* isolates. We first performed a GWAS using MLM and MLMM models and found that MLM (**Supplementary Data S3–5**) and MLMM (**Supplementary Data S6–8**) provided similar results; however, MLMM detected additional significant associations for leaf and stem rust diseases. Both models detected SNPs associated with stripe and stem rust resistance in physical regions of the cloned genes *Yr15* and *Sr13*. Thus, gene-specific markers *WTK1_Kin1* (*Yr15*) and *CNL13* (*Sr13*) were added to the set of genetic markers used for the final GWAS analysis using MLMM.

**Figure 3:**
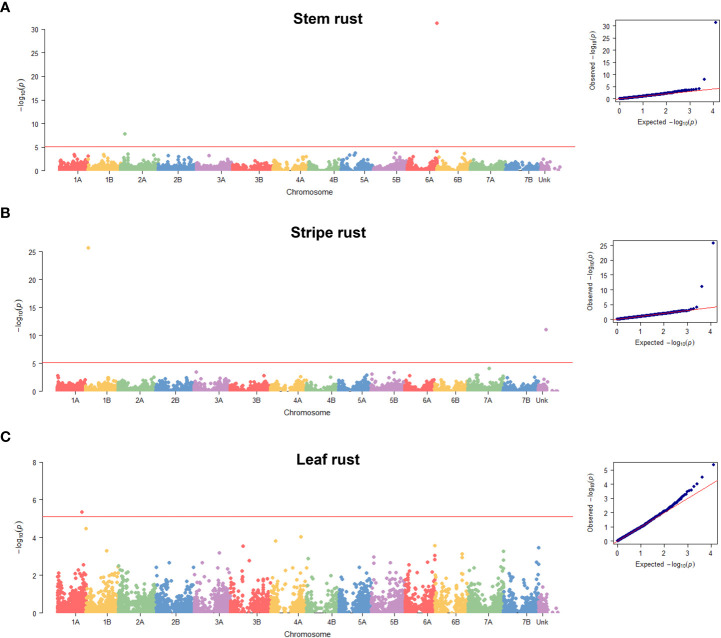
Manhattan plots of the SNPs. Plots displayed across the 14 wheat chromosomes of Svevo v1 (Maccaferri et al., 2019) and the unknown pseudo-chromosome that collates the unassembled contigs that indicate the SNPs associated with resistance to stem rust DCB race **(A)**, stripe rust W001 race **(B)** and leaf rust race 1 **(C)**. Quantile-quantile plots are displayed on the right of each Manhattan plot. Red horizontal line represents the Bonferroni threshold of α = 0.05/*n* where *n*=6,412.

The final GWAS revealed five significant QTNs associated with resistance to one of the three rusts (**Figure 3**; **Table 1** and **Supplementary Data S9**–**11**). Of them, *CNL13_Sr13* and *GENE-1196_146* markers associated with resistance to stem rust localized on chromosomes 6A and 2A, respectively (**Supplementary Data S9**); *Excalibur_s115495_117* marker associated with leaf rust resistance was mapped to chromosome 1A (**Supplementary Data S11**); and *WTK1_Kin1_Yr15* and *Tdurum_contig49575_1237* markers associated with stripe rust resistance were detected on chromosome 1B and on chromosome “unknown” of the Svevo v1 genome assembly (Maccaferri et al., 2019), the latter is the result of the collation of unassembled contigs (**Supplementary Data S10**). To decipher the probable chromosomal position of *Tdurum_contig49575_1237*, we performed a BLAST search using marker probes as queries. The best BLAST hit for this marker was on chromosome 7B for both, Zavitan v2 (Chr7B:735903254…735903205) (Zhu et al., 2019) and Chinese Spring v1 (chr7B:718358622…718358671) (IWGSC, 2018) wheat genome assemblies. We also determined that this marker localizes genetically to chromosome 7B on a tetraploid wheat consensus map (Maccaferri et al., 2015). Taken together, these independent data indicate the likelihood of localization of this marker on chromosome 7B.

**Table 1:** Marker-trait associations for resistance to rust diseases in the diversity panel obtained using the MLMM model.

	Stem rust		Stripe rust		Leaf rust
QTN	*CNL13_Sr13*	*GENE-1196_146*	*WTK1_Kin1_Yr15*	*Tdurum_contig 49575_1237*	*Excalibur_s115495_117*
Chr*	6A	2A	1B	Un(7B^#^)	1A
Position*	611710998	91356805	57212786	164318835 (235903205^#^)	497569466
MAF	0.1	0.14	0.09	0.11	0.21
P-value	5.82E-32	1.34E-08	2.21E-26	9.2E-12	4.51E-06
Bonferroni-adjusted P-value	0	8.59E-05	1.42E-22	5.90E-08	2.89E-02
Kruskal-Wallis allelic test *p*-value	<2.2E-16	<2.2E-16	<2.2E-16	1.02E-05	<2.2E-16
% genetic variance explained	49.83	22.16	47.22	15.36	45.54
Favorable allele	A	A	A	B	A
WEW with favorable allele (%)			20.45	2.84	5.65
DEW with favorable allele (%)	13.74	19.08			62.60
DW_LR with favorable allele (%)	20.14	26.62	1.44	32.37	
Co-located gene(s)	*Sr13*		*Yr15*	*Yr67*	

^*^Chromosome position provided according to Svevo v1 (Maccaferri et al., 2019) assembly.

^#^Chromosome position according to Zavitan v2 (Zhu et al., 2019) assembly.

### Candidate gene analysis

#### Stem rust

Two QTNs were detected for stem rust infection type against race DCB. The *Sr13* gene-specific marker *CNL13* (Zhang et al., 2017) located on chromosome arm 6AL, was identified as associated with resistance to stem rust (**Figure 3**; **Table 1**), and resistance phenotype correlated with the presence of a functional allele (**Supplementary Figure S6**). The resistant allele was detected in DEW and DW_LR of the diversity panel, but was absent in WEW (**Table 1**; **Supplementary Figure S6** and **Supplementary Data S13**).

The second stem rust QTN is located on chromosome arm 2AS, and the only gene previously mapped to this chromosome arm is *Sr38*. However, it originates from *Aegilops ventricosa*, thus, its presence in our diversity panel at a frequency >5% is unlikely. Box plots show a phenotypic separation of genotypes carrying resistance-related allele compared with those carrying susceptibility-related allele (**Supplementary Figure S6**). The Svevo v1 annotation (Maccaferri et al., 2019) contains six high confidence genes within the *GENE-1196_146* QTN confidence interval (marker position ± LD decay) (**Supplementary Data S12**), none of which belong to the well-known disease resistance gene analogues such as nucleotide-binding and leucine-rich repeat (NLR), protein kinase, tandem kinase protein (TKP) for examples (Kourelis and van der Hoorn, 2018; Klymiuk et al., 2021). The resistance-related allele was detected in DEW and DW_LR genotypes of the diversity panel, but not in WEW (**Supplementary Figure S6**; **Supplementary Data S13**).

#### Stripe rust

Two MTAs were detected for stripe rust resistance. The *WTK1_Kin1* is a marker derived from the cloned *Yr15* gene, which differentiates the functional and non-functional alleles of *Wheat Tandem Kinase 1* (*WTK1*) (Klymiuk et al., 2019). An analysis of correspondence between the resistance phenotype and *Wtk1* functional allele (Klymiuk et al., 2019) revealed good alignment (**Supplementary Data S13**). *Yr15* was detected in 8.37% of the genotypes in the diversity panel, predominantly in the WEW accessions; however, the DW_LR accessions Simhon and Uzan from Israel also showed the presence of the *Wtk1* functional allele (**Supplementary Figure S6**; **Supplementary Data S13**).

The second MTA identified for stripe rust is the *Tdurum_contig49575_1237* QTN, which as we noted above likely localizes to chromosome 7B (long arm). Of the officially designated stripe rust resistance genes, six were previously mapped to chromosome 7BL/7B. Of them, *Yr59*, *Yr52*, and *Yr39* represent APR genes that would not have been detected in our seedling tests. The remaining three are the race-specific ASR resistance genes: *Yr6*, *Yr2*, and *Yr67* (McIntosh et al., 2020). The *Pst* race W001 used in the current study is virulent on *Yr2* and *Yr6* (**Supplementary Table S2**) and, as such, they could not account for the detected QTN. Finally, the physical position of *Yr67* on chromosome arm 7BL of the Chinese Spring reference genome (chr7B:716,966,290…721,082,714) (Bariana et al., 2022) overlaps with the position of *Tdurum_contig49575_1237* QTN in this assembly. Therefore, *Yr67* could be present in our diversity panel and could be the casual gene detected by this QTN on chromosome 7B. The Zavitan annotation (Zhu et al., 2019) contains 16 genes within the confidence interval of the *Tdurum_contig49575_1237* QTN, including some that encode proteins with domains commonly found in disease resistance proteins, such as protein kinases and NLRs (**Supplementary Data S12**), which are worthy of investigation as potential candidate genes. The resistance allele was detected predominantly in the DW_LR germplasm from all geographic regions and a few WEW genotypes, while none the DEW genotypes possessed the favorable allele (**Supplementary Figure S7**; **Supplementary Data S13**).

#### Leaf rust

The single QTN *Excalibur_s115495_117* was detected for leaf rust on chromosome 1A (**Table 1**). The wheat gene catalogue (McIntosh et al., 2020) lists only *Ae. peregrina* gene *Lr59* for chromosome arm 1AL, which is not likely present in our diversity panel. Other studies have reported QTLs for leaf rust resistance on chromosome arm 1AL, but the reported positions do not overlap with the QTL identified herein (Aoun et al., 2016; Gao et al., 2016; Sapkota et al., 2019; Fatima et al., 2020). The Svevo v1 annotation (Maccaferri et al., 2019) contains 16 high-confidence genes within the *Excalibur_s115495_117* QTN confidence interval (**Supplementary Data S12**); however, none share signatures of domains related to plant immunity. Interestingly, the resistance-associated allele was detected only in DEW and WEW accessions (**Supplementary Figure S6**; **Supplementary Data S13**) with clear phenotypic differences between genotypes that carry the favorable allele compared with those carrying the alternate allele for DEW, but not WEW (**Supplementary Figure S6**).

## Discussion

Diversity panels are a useful resource to detect alleles currently untapped in breeding, and a number of studies have reported novel disease resistance sources within wheat diversity panels (Bulli et al., 2016; Prins et al., 2016; Chao et al., 2017; Tessmann et al., 2019; Fatima et al., 2020; Miedaner et al., 2020). Panels consisting of multi-subspecies may also capture evolutionary history of the crop and are used to track evolutionary changes between species or subspecies, for example, the domestication process (Tian et al., 2009; Scott et al., 2019; Mazzucotelli et al., 2020; Parker et al., 2020). In the current research, we used a tetraploid wheat diversity panel that includes three wheat subspecies representing different stages of domestication: exclusively wild non-domesticated (WEW), primary level of domestication (DEW), and durum landraces (DW_LR). This panel is comprised of genotypes that are genetically diverse, as was demonstrated by our 90K data analysis, and that originated from various geographic regions. Geographic origin influences crop evolution in several ways through diverse climatic conditions, cultural practices, and historical events in different parts of the globe. LD decay in this panel extended to less than 1 Mb, similar to another panel comprised of diverse tetraploid wheat accessions (Mazzucotelli et al., 2020). This level of LD is sufficiently small to provide efficient markers for marker-assisted selection/introgression of the identified QTNs.

In terms of population structure, our analyses showed that our panel consists of three subpopulations: 1) DW_LR, 2) WEW + part of DEW (41 accessions), and 3) Ethiopian DEW (90 accessions). The genetic separation of DEW of Ethiopian origin from all other DEW genotypes is, as supported by previous studies shown, showed that Ethiopian-origin wheat is genetically distinct from other wheat genotypes (Kidane et al., 2019; Mazzucotelli et al., 2020). On the other hand, the grouping of several accessions of DEW with the northern WEW accessions from Turkey, Iran, and Iraq was surprising considering that they are systematically distinct. These results support the previously stated hypothesis that wheat was domesticated in the northern part of WEW natural growing region (Luo et al., 2007; Özkan et al., 2011), and thus, DEW genotypes still share some genetic relatedness with those northern WEW populations. This may be an interesting direction for future evolution-related studies using this diversity panel.

In our diversity panel, we have detected MTAs for infection type for each of the three wheat rust diseases. Preliminary analysis showed MTAs for stripe and stem rusts on chromosomes 1B and 6A, respectively. We postulated that these may be associated with the previously cloned genes *Yr15* (1B) and *Sr13* (6A). The genotyping of the entire panel with *Yr15* and *Sr13* gene-specific markers confirmed their association with the two QTNs (**Table 1**). Moreover, based on the overlap of the QTN significance interval with the *Yr67* mapping interval, it is also possible that the stripe rust resistance localized to chromosome 7B may be associated with *Yr67*, but the causal effect remains to be confirmed. At the same time, the *Lr* locus at chromosome arm 1AL and *Sr* locus at chromosome arm 2AS harbor previously undescribed race-specific resistance genes because the QTN confidence intervals for these loci do not overlap with any officially designated genes. The detected associations are race-specific and represent genes/QTLs providing resistance to the rust races used in the current study. Our diversity panel may harbor other race-specific rust resistance genes against other races not tested herein. Testing of this diversity panel with other rust races may reveal other resistance genes/QTLs as reported for the Ethiopian Durum Wheat panel tested with four *Pst* races that possess diverse virulence profiles (Tene et al., 2022).

There was no overlap between any of the QTN loci, which was expected because all multi-disease resistance genes known to date are APR genes (Chen and Kang, 2017) and these could not be detected in our seedling tests. Discovery of APR genes could be achieved by evaluating severity in field experiments (Fatima et al., 2020; Tene et al., 2022), which is a proposed future direction for exploring the potential and exploiting the value of this diversity panel. Expansion to include traits other than disease resistance, such as morphological traits, is also anticipated to lead to interesting and beneficial outcomes with possible application in wheat breeding programs.

For all three diseases, proportionately more DW_LR accessions were resistant compared with WEW accessions, indicating that, even at the landrace level, resistance is available to support breeding. At the same time, our analysis showed the importance of DEW as a source of resistance to all three wheat rust diseases, and introgression to modern cultivars is expected to enrich the resistance gene pool of cultivated durum wheat. Among DEW accessions, the lines of Ethiopian origin are particularly interesting as was highlighted in previous studies (Kidane et al., 2019; Mazzucotelli et al., 2020). In our experiments, the percentage of Ethiopian DEW lines with resistance to stripe and leaf rust races was much higher than that of DEW genotypes of other geographic origin, but in contrast, they lacked stem rust resistance. Taking into account that all three wheat rust pathogens are detected in all wheat growing areas, and that they require similar conditions for pathogen development, there seems to be no clear reason explaining this host-pathogen co-evolution in Ethiopia (Klymiuk et al., 2019) for stripe and leaf rusts compared to stem rust. Thus, the observed picture may be the result of the use of the specific pathogen races in the current study. Once more, testing with multiple races of the three rust species could shed light on this observation.

While we were able to detect at least two potentially novel QTNs associated with rust resistance in this study, we did note that several accessions of all three subspecies were resistant to one or more of the rust races evaluated but did not carry the favorable alleles. Other resistance genes are likely present in our diversity panel but were simply not detected because of their low frequency, their small effect or underrepresentation of their genomic regions due to genotyping resolution. Indeed, we noted that 3,757 (representing nearly 1/3 of the polymorphic markers) were present at a frequency of <5% in the population. Low-frequency variants often do not pass statistical significance thresholds in GWAS studies; thus their effects are often missed or biased. This is likely the case in our GWAS panel, and we are examining accessions lacking the QTN-associated resistances reported here but expressing near immunity to all three rust pathogens (e.g., DEW PI94635) or at least two rust pathogens (e.g., WEW PI478694 and DEW PI352362) (**Supplementary Data S13**).

To conclude, we have assembled a diverse panel of 447 genotypes from three wheat subspecies representing a range of improvement statuses and diverse geographic origins. The GWAS analyses detected the following statistically significant associations: two for stem rust on chromosomes 6A and 2A; two for stripe rust on chromosomes 1B and 7B; one for leaf rust on chromosome 1A. Two of the detected associations represent the previously cloned *Sr13* and *Yr15* genes and one overlap with the *Yr67* locus, while the other two were located at positions where no officially designated resistance genes had been reported. The developed diversity panel could serve as a good resource for future association-mapping studies of other traits of interest and for evolutionary studies.

## Data availability statement

The original contributions presented in the study are included in the article/**Supplementary Material**. Further inquiries can be directed to the corresponding author.

## Author contributions

VK and CP contributed to conception and design of the study. VK maintained seed collection and performed wheat rust severity phenotyping. VK, TH, AF and AN performed data analysis. JE and KW produced wheat 90K array data. SH, TK, and RB-D suggested and/or provided some of the key germplasm. SC and CP provided financial support and project supervision as part of the 4D Wheat Project. VK and TH drafted the manuscript with input from all co-authors. All authors contributed to the article and approved the submitted version.
