# Supplementary material for: Genetic architecture of rust resistance in a wheat (Triticum turgidum) diversity panel
Source: Front Plant Sci. 2023 Mar 14;14:1145371. doi: 10.3389/fpls.2023.1145371 (PMC10043469; doi:10.3389/fpls.2023.1145371)
Supplement: Supplementary file 1 [file DataSheet_1.pdf]

Supplementary Material

Genetic architecture of rust resistance in a wheat (*Triticum turgidum*) diversity panel

Valentyna Klymiuk[1], Teketel Haile[1], Jennifer Ens[1], Krystalee Wiebe[1], Amidou N'Diaye[1], Andrii Fatiukha[1], Tamar Krugman[2], Roi Ben-David[3], Sariel Hübner[4], Sylvie Cloutier[5,6] and Curtis J. Pozniak[1]*

*** Correspondence:** Curtis J. Pozniak: curtis.pozniak@usask.ca

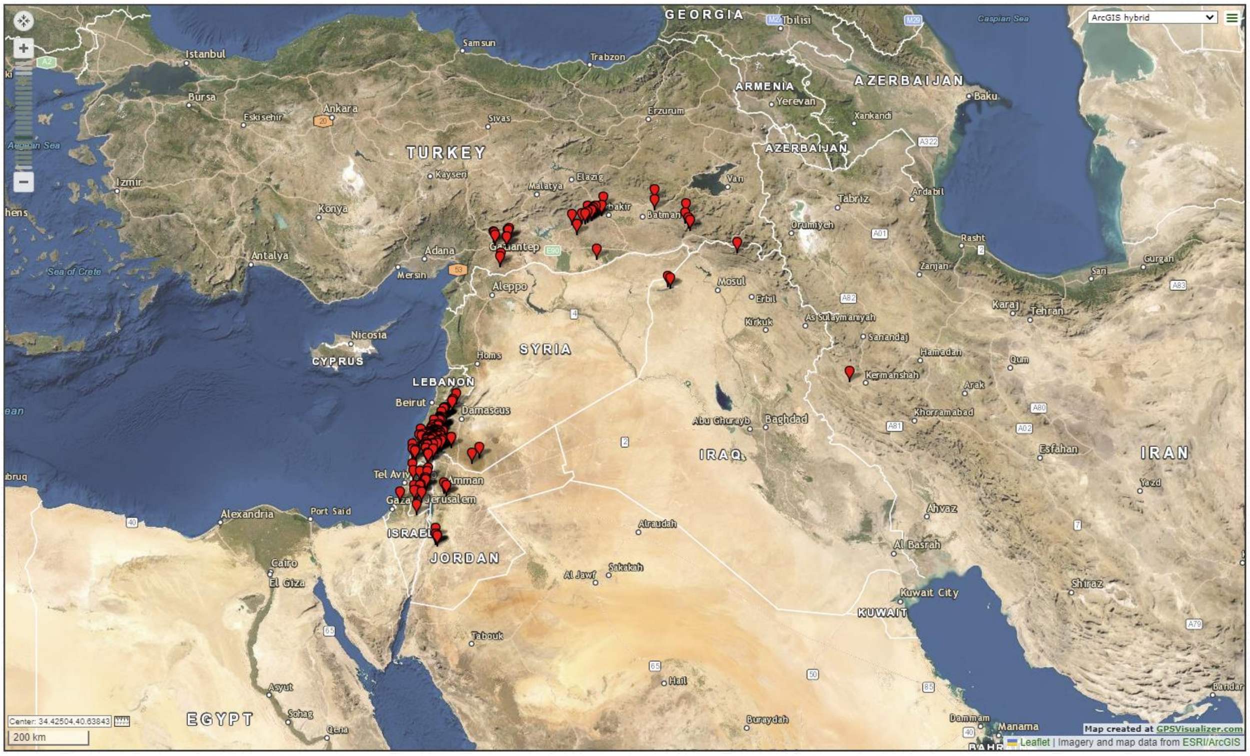

Supplementary Figure S1. Geographic distribution of 177 accessions of wild emmer wheat (*Triticum turgidum* ssp. *dicoccoides*) from seven countries of the Fertile Crescent region.

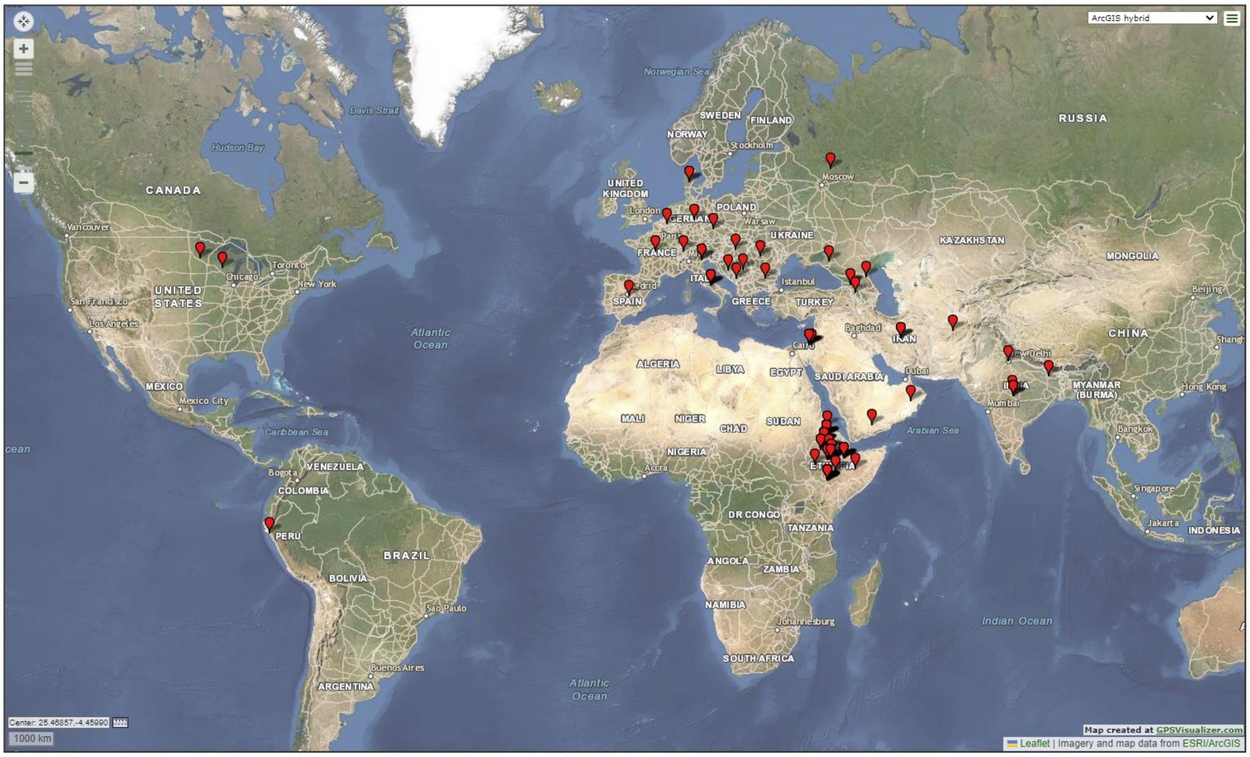

Supplementary Figure S2. Geographic distribution of 131 accessions of domesticated emmer wheat (*T. turgidum* ssp. *dicoccum*) from 27 countries

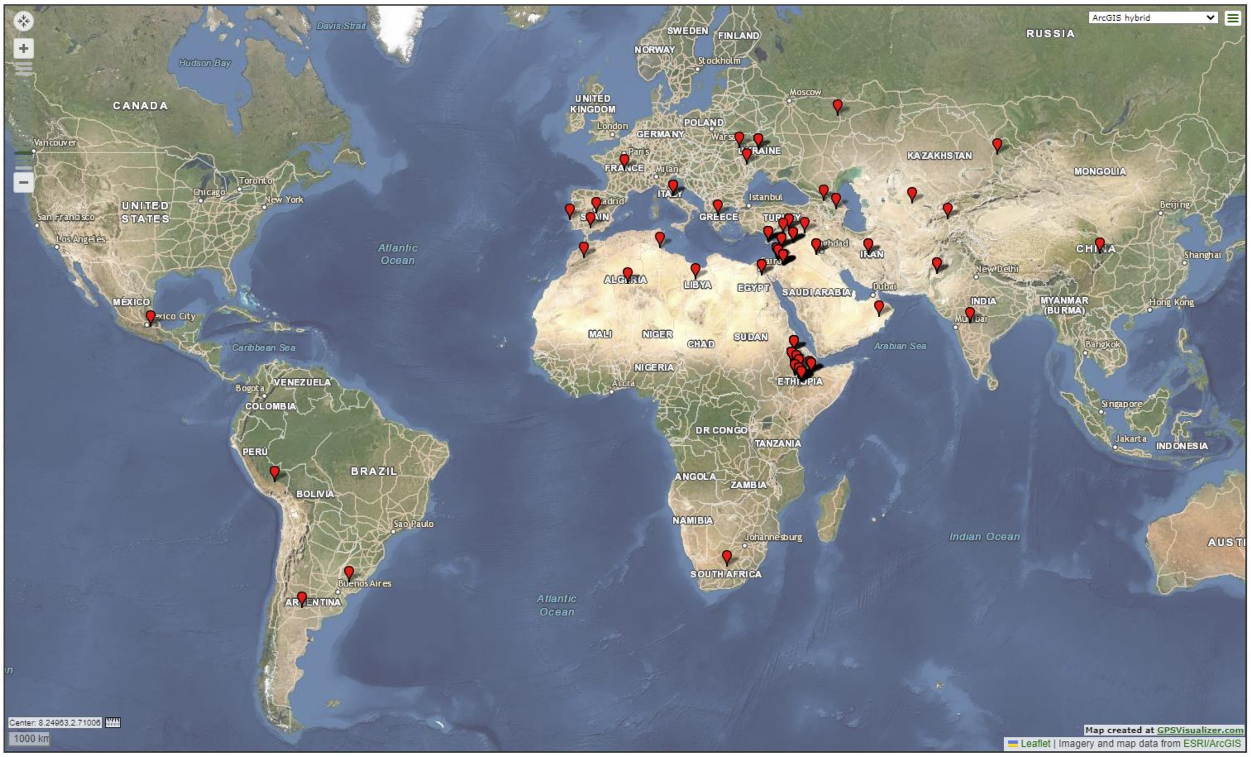

Supplementary Figure S3. Geographic distribution of 139 accessions of durum wheat (*T. turgidum* ssp. *durum*) landraces from 37 countries.

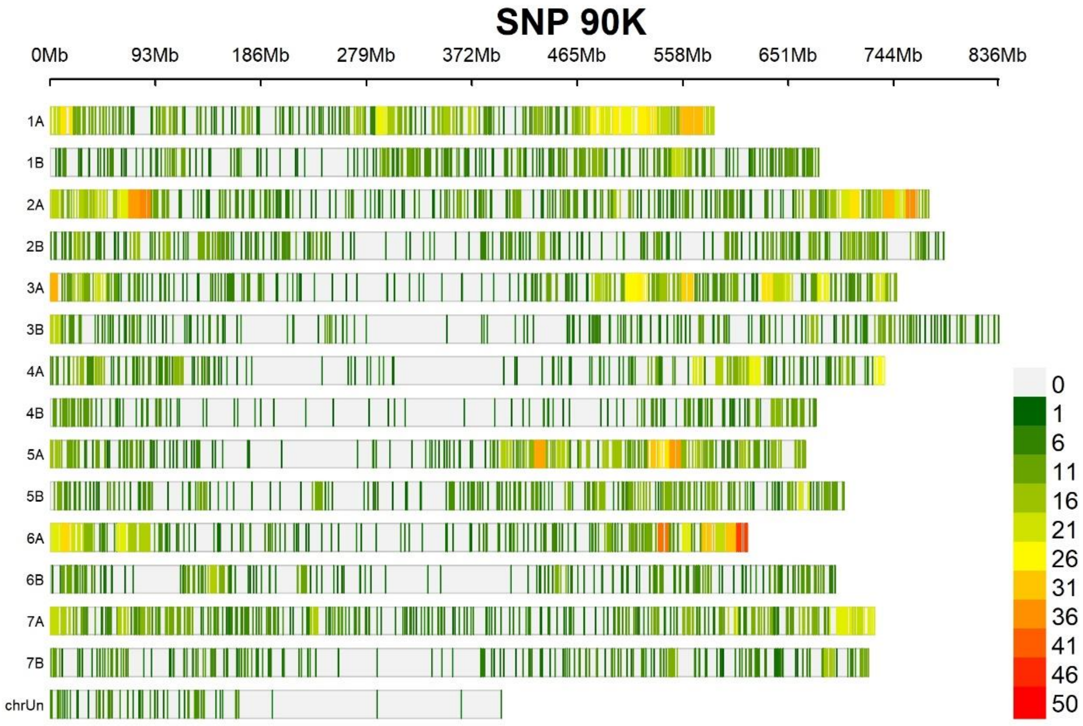

Supplementary Figure S4. Distribution of the 6,410 markers selected for the MLMM analysis along the 15 pseudomolecules of tetraploid wheat reference genome Svevo v1 (Maccaferri et al., 2019) based on the physical positions. Color code is marker density in 10 Mbp.

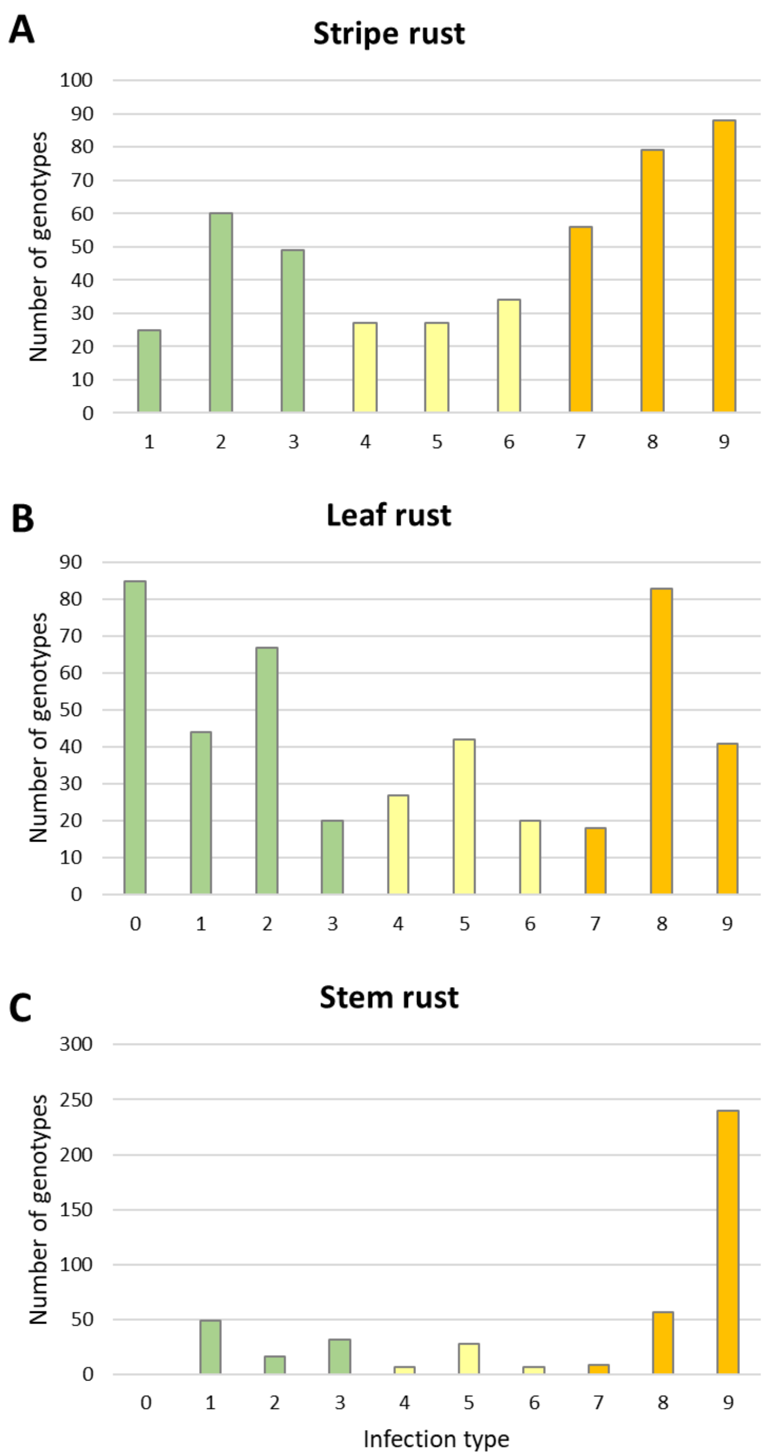

Supplementary Figure S5. Distributions of the accessions based on their infection type following inoculation with (**A**) stripe rust, (**B**) leaf rust, and (**C**) stem rust in the diversity panel. Infection types on X axis are rounded BLUP scores. Color codes represent interpretation of phenotyping scales: green – resistance, yellow – moderate resistance, orange – susceptibility.

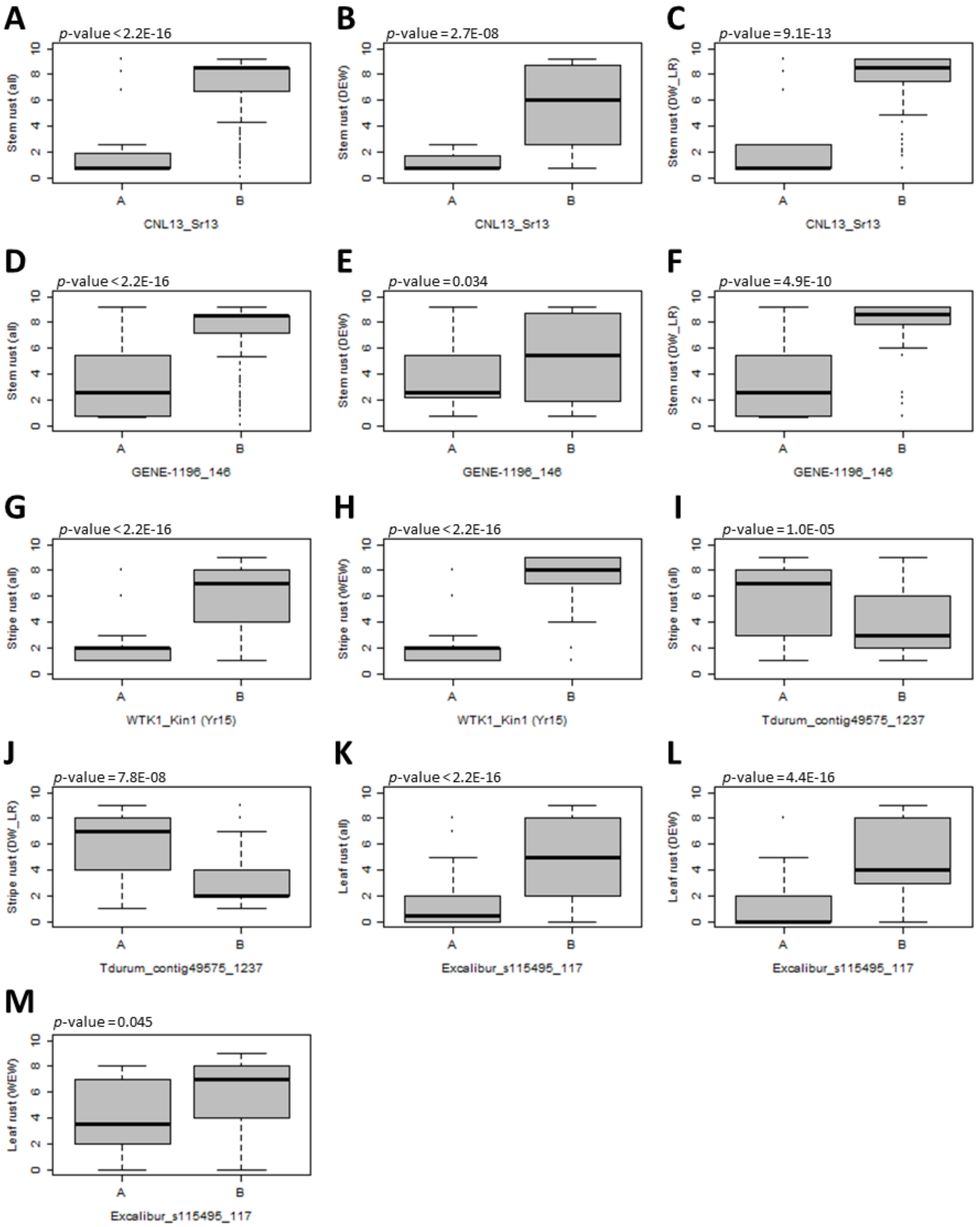

Supplementary Figure S6. Boxplots that contrast the two alleles of markers associated with stem (**A-F**), stripe (**G-J**) and leaf rust resistance (**K-M**): *CNL13_Sr13* entire panel (**A**), DEW subgroup (**B**) and DW_LR subgroup (**C**); *GENE-1196_146* entire panel (**D**), DEW subgroup (**E**) and DW_LR subgroup (**F**); *WTK1_Kin1* (*Yr15*) entire panel (**G**), and WEW subgroup (**H**); *Tdurum_contig49575_1237* entire panel (**I**) and DW_LR subgroup (**J**); *Excalibur_s115495_117* entire panel (**K**), DEW subgroup (**L**) and WEW subgroup (**M**). Infection types on X axis are rounded BLUP scores. p-values represent Kruskal-Wallis non-parametric test applied as an allelic test.

Supplementary Table S1. Rust diseases scores of the checks used for phenotyping of the 4D 90K panel. Numbers are infection type scores. R – resistant, MR – moderate resistant and S – susceptible responses.

**Genotype**	**Taxonomy**	**Stripe rust score**[#]	**Leaf rust score***	**Stem rust score**
AAC Cabri	*T. turgidum* ssp. *durum*	2 (R)	; 1 (R)	; (R)
AAC Tisdale	*T. aestivum*	6-8 (MR-S)	0 ; (R)	0 ; (R)
AC Barrie	*T. aestivum*	7-9 (S)	; (R)	0 ; (R)
AC Navigator	*T. turgidum* ssp. *durum*	8 (S)	; 1 2 (R-MR)	; 1 (R)
Avocet S	*T. aestivum*	9 (S)	; (R)	; 1 (R)
Avocet+*Yr15*	*T. aestivum*	0-1 (R)	; (R)	; 1 (R)
Avocet+*Yr5*	*T. aestivum*	1 (R)	; (R)	; 1 (R)
Brigade	*T. turgidum* ssp. *durum*	4-5 (MR)	; 1 (R)	; (R)
BW1085	*T. aestivum*	7-8 (S)	; (R)	0 ; (R)
Cardale	*T. aestivum*	6-7 (MR-S)	0 ; (R)	0 (R)
CDC Defy	*T. turgidum* ssp. *durum*	2-3 (R)	1 2 (R-MR)	; (R)
CDC Desire	*T. turgidum* ssp. *durum*	2-3 (R)	; 1 2 (R-MR)	; (R)
CDC Landmark	*T. aestivum*	7-8 (S)	0 ; (R)	0 (R)
CDC Precision	*T. turgidum* ssp. *durum*	2-4 (R-MR)	; 1 (R)	; 1 (R)
CM-82036	*T. aestivum*	6-9 (MR-S)	; (R)	; 1 (R)
Columbus	*T. aestivum*	7-9 (S)	; 1 (R)	0 ; (R)
DT1020	*T. turgidum* ssp. *durum*	2-4 (R-MR)	; (R)	; (R)
FHB37	*T. aestivum*	6-8 (MR-S)	0 ; (R)	0 ; (R)
Glenlea	*T. aestivum*	6-8 (MR-S)	; (R)	0 ; (R)
Glenn	*T. aestivum*	7-8 (S)	0 ; (R)	0 ; (R)
Hoffman	*T. aestivum*	7-9 (S)	0 ; (R)	2 (MR)
Kronos	*T. turgidum* ssp. *durum*	8-9 (S)	1 2 (R-MR)	1 (R)
McKenzie	*T. aestivum*	7-9 (S)	; (R)	0 ; (R)
McNair	*T. aestivum*	8-9 (S)	; (R)	4 (S)
Morse	*T. turgidum* ssp. *durum*	7-9 (S)	; 1 (R)	; (R)
PI 487260 (*Yr84*)	*T. turgidum* ssp. *dicoccoides*	0-2 (R)	4 (S)	4 (S)
Ruta	*T. aestivum*	9 (S)	; (R)	2-3 (MR-S)
Strongfield	*T. turgidum* ssp. *durum*	2-3 (R)	; (R)	; (R)
Thatcher	*T. aestivum*	8-9 (S)	4 (S)	4 (S)
Toing3	*T. aestivum*	9 (S)	; (R)	; (R)
Westbred 881	*T. turgidum* ssp. *durum*	8-9 (S)	; 1 2 (R-MR)	; 1 (R)

[#]McNeal scale – 0 to 9 infection types (McNeal et al., 1971).
[*]Stakman scale – 0 to 4 infection types (Stakman et al., 1962).

Supplementary Table S2. Virulence profiles of wheat stem, stripe and leaf rust races used in this study.

**Disease**	**Name of isolate/race**	**Pathotype**	**Virulence profile**	**Reference**
Stripe rust	W001	NA*	*YrA*, *Yr2*, *Yr6*, *Yr7*, *Yr8*, *Yr9*, *Yr17*, *Yr25*, *Yr27*, *Yr28*, *Yr29*, *Yr31*, *YrSu*	Brar et al., 2018
Leaf rust	race 1	BBBD	*Lr14a*, *Tc*, *LrCen*	Cuomo et al., 2017
Stem rust	DCB	TRRTF	*Sr5*, *Sr21*, *Sr9e*, *Sr7b*, *Sr11*, *Sr6*, *Sr9g*, *Sr36*, *Sr9b*, *Sr17*, *Sr9a*, *Sr9d*, *Sr10*, *SrTmp*, *Sr38*, *SrMcN*	Tested in this study

*NA – not applicable

Supplementary Table S3. Phenotypic responses of the *T. turgidum* sub-species of the diversity panel to three rust diseases.

Sub-species	Genotypes	Resistant	Moderate resistant	Susceptible	Total
**Stem rust (DCB)**					
**DEW**	No.	57	20	54	131
	%	43.5	15.3	41.2	100
**DW_LR**	No.	37	10	92	139
	%	26.6	7.2	66.2	100
**WEW**	No.	4	12	160	176
	%	2.3	6.8	90.9	100
**Total**	No.	98	42	306	446
	%	22.0	9.4	68.6	100
**Stripe rust (W001)**					
**DEW**	No.	41	37	53	131
	%	31.3	28.2	40.5	100
**DW_LR**	No.	49	26	62	137
	%	35.8	19.0	45.2	100
**WEW**	No.	44	25	108	177
	%	24.9	14.1	61.0	100
**Total**	No.	134	88	223	445
	%	30.1	19.8	50.1	100
**Leaf rust (race 1)**					
**DEW**	No.	99	13	19	131
	%	75.6	9.9	14.5	100
**DW_LR**	No.	74	32	33	139
	%	53.3	23.0	23.7	100
**WEW**	No.	43	43	90	176
	%	24.4	24.4	51.2	100
**Total**	No.	216	88	142	446
	%	48.4	19.8	31.8	100

Supplementary Table S4. Phenotypic responses of DEW genotypes of different origin to three rust diseases.

DEW origin	Genotypes	Resistant	Moderate resistant	Susceptible	Total
**Stem rust (DCB)**					
**Ethiopian**	No.	31	12	39	82
	%	37.8	14.6	47.6	100
**All other**	No.	26	8	15	49
	%	53.1	16.3	30.6	100
**Stripe rust (W001)**					
**Ethiopian**	No.	37	24	21	82
	%	45.1	29.3	25.6	100
**All other**	No.	4	13	32	49
	%	8.2	26.5	65.3	100
**Leaf rust (race 1)**					
**Ethiopian**	No.	71	4	7	82
	%	86.6	4.9	8.5	100
**All other**	No.	28	9	12	49
	%	57.1	18.4	24.5	100

References:

Brar, G.S., Ali, S., Qutob, D., Ambrose, S., Lou, K., Maclachlan, R., et al. (2018). Genome re-sequencing and simple sequence repeat markers reveal the existence of divergent lineages in the Canadian *Puccinia striiformis* f. sp. *tritici* population with extensive DNA methylation. Environmental Microbiology 20 (4). doi:10.1111/1462-2920.14067.

Cuomo, C.A., Bakkeren, G., Khalil, H.B., Panwar, V., Joly, D., Linning, R., et al. (2017). Comparative analysis highlights variable genome content of wheat rusts and divergence of the mating loci. G3: Genes, Genomes, Genetics 7 (2). doi:10.1534/g3.116.032797.

Maccaferri, M., Harris, N.S., Twardziok, S.O., Pasam, R.K., Gundlach, H., Spannagl, M., et al. (2019). Durum wheat genome highlights past domestication signatures and future improvement targets. Nature Genetics 51 (5). doi:10.1038/s41588-019-0381-3.

McNeal, F.H., Konzak, C.F., Smith, E.P., Tate, W.S., Russell, T.S. (1971). A uniform system for recording and processing cereal research data. U.S. Dept. Agric., Agric. Res. Serv., ARS 34-121, 42 pp.

Stakman, E.C., Stewart, D.M., Loegering, W.Q. (1962). Identification of physiologic races of *Puccinia graminis* var. *tritici*. US Department of Agriculture Agricultural Research Service E-617, Washington DC.
